# First use of gene therapy to treat growth hormone resistant dwarfism in a mouse model

**DOI:** 10.1038/s41434-022-00313-w

**Published:** 2022-02-01

**Authors:** Kian Chuan Sia, Shu Uin Gan, Siti Humairah Mohd Rodhi, Zhen Ying Fu, John J. Kopchick, Michael J. Waters, Kok Onn Lee

**Affiliations:** 1 Department of Surgery, National University of Singapore, Singapore, Singapore; 2 Edison Biotechnology Institute, Ohio University, Athens, OH USA; 3 Institute for Molecular Bioscience, The University of Queensland, St. Lucia, QLD Australia; 4 Department of Medicine, National University of Singapore, Singapore, Singapore

**Keywords:** Targeted gene repair, Genetic vectors

## Abstract

The only treatment tested for growth hormone receptor (GHR) defective Laron Syndrome (LS) is injections of recombinant insulin-like-growth factor 1 (rhIGF1). The response is suboptimal and associated with progressive obesity. In this study, we treated 4–5-week-old Laron dwarf mice (GHR−/−) with an adeno-associated virus expressing murine GHR (AAV-GHR) injection at a dose of 4 × 10^10^ vector genome per mouse. Serum growth hormone (GH) levels decreased, and GH-responsive IGF1, IGF binding protein 3 (IGFBP3) and acid labile subunit (ALS) increased. There was a significant but limited increase in body weight and length, similar to the response to rhIGF1 treatment in LS patients. All the major organs increased in weight except the brain. Our study is the first to use gene therapy to treat GH-receptor deficiency. We propose that gene therapy with AAV-GHR may eventually be useful for the treatment of human LS.

## Introduction

Laron first described the syndrome of short stature with growth hormone (GH) insensitivity, and elevated GH with undetectable insulin-like-growth factor 1 (IGF1) in 1966 [1–3]. Various trials have attempted to treat these patients with the downstream growth effector of GH, recombinant IGF1 [4–6]. Recombinant IGF1 (rhIGF1) has a much shorter half-life [7] than endogenous IGF1, which is normally bound to IGF binding protein 3 (IGFBP3) and acid labile subunit (ALS) [8, 9]. Therefore, daily [10] and subsequently twice daily [4–6] injections of rhIGF1 were given to attempt to increase treatment efficacy [11]. All these studies with IGF1 were stopped for various reasons, including poor efficacy, progressive obesity [12], commercial and other reasons [13, 14].

We have attempted to use a strategy of increasing IGF1 in an animal model by replacing the disabled GH-receptor (GHR) gene using a viral vector with a liver-specific transcriptional regulatory sequence, since this organ is the major source of circulating IGF1. This approach should increase circulating IGF1 without any change in GHR in tissues other than the liver. It would provide additional information on the reasons for the limited results in the human trials. The adeno-associated virus (AAV) has recently been approved for the treatment of various human genetic diseases by the Food and Drug Administration and the European Medicines Agency (e.g., Luxturna, Strimvelis, Zolgensma, Yescarta, Kymriah and Glybera) [15]. The delivered gene is maintained mainly episomally [16] with persistent gene expression, making the AAV vector an excellent gene delivery tool for treatment of some inherited human diseases [15]. Furthermore, there is persistent gene expression for more than 10 years following a single dose of vector administration, making AAV gene therapy an attractive treatment with potentially significant therapeutic outcomes [17].

To our knowledge, this is the first reported pre-clinical gene therapy treatment of an established model of the Laron Syndrome (LS), i.e., the GHR−/− or “knockout” mice [18] using an AAV8 vector directing expression of mouse GHR (mGHR) to the liver. We asked if expression of GHR driven by a liver-specific promoter/enhancer would (1) induce the expression and secretion of IGF1; (2) increase the expression of IGFBP3 and thus increase the half-life of IGF1; and (3) increase expression of ALS to further increase the stability and bioavailability of circulating IGF1. We aimed to investigate if a single dose of AAV8-mediated liver expression of mGHR was sufficient to treat Laron dwarf mice, as a proof of concept for development of a treatment for patients with LS.

## Materials and methods

### Plasmid DNA constructs

Mouse GHR expressing plasmid pAAV-HLP-mGHR was generated by swapping the human insulin gene (hINSco) in previously generated pAAV-HLP-hINSco [19] with a mGHR PCR fragment using NotI and XhoI sites. The mGHR PCR fragment was amplified from cloning vector MG50043-M (Sino Biological, Beijing, China) containing mouse growth hormone receptor/GHR/GHBP transcript variant 1 gene ORF cDNA clone using forward primer with introducing NotI site: 5’-ATAAGAATGCGGCCGCACCATGGATCTTTGTCAGGTCTTC and reverse primer with introducing XhoI site: 5’-CCGCGCTCGAGCTACTGCATGATTTTGTTCAGTTG. The control plasmid pAAV-HLP-Luc was previously generated [20].

### Cell transfection

1 × 10^6^ HepG2 cells were seeded in each well of 6‐well plates. Cells were transfected with 2500 ng of pAAV‐HLP‐Luc or pAAV‐HLP-mGHR plasmids using Lipofectamine 3000 reagent (Thermo Fisher Scientific, Waltham, MA, USA) according to manufacturer’s instruction. The cells were washed with PBS and pelleted down for RNA and protein extraction.

### RT-PCR gel

Total RNA from HepG2 transfected cells was extracted using RNeasy mini kit according to manufacturing protocol (QIAGEN, Valencia, CA, USA). One µg of total RNA was converted to cDNA using random hexamer primers (Integrated DNA Technologies, Coralville, IA, USA), oligo-dT primers (Integrated DNA Technologies) and RNase H Minus Reverse Transcriptase (Thermo Fisher Scientific) according to the manufacturer’s protocol. PCR was performed for 27 cycles with GoTaq polymerase (Promega, Madison, WI, USA) using primers designed against the mGHR [21] (forward primer 5’- GATTTTACCCCCAGTCCCAGTTC-3’ and reverse primer 5’-GACCCTTCAGTCTTCTCATCCACA-3’) and mouse 18S rRNA (forward primer 5’-CCTGCGGCTTAATTTGACTC-3’ and reverse primer 5’-CGCTGAGCCAGTCAGTGTAG-3’) before subjected to agarose gel analysis.

### Western blot

HepG2 transfected cells were lysed in 300 µl of RIPA lysis buffer (0.15 M NaCl, 0.05 M Tris pH 7.5, 0.1% SDS, 0.5% sodium deoxycholate, 1% NP-40) with protease inhibitor cocktail P8340 (Sigma Aldrich, St. Louis, MO, USA). 10 µg of proteins were separated by sodium dodecyl sulfate polyacrylamide gel electrophoresis and blotted to polyvinylidene fluoride membrane using wet-transfer method. Thereafter, it was immunoblotted with mouse anti-GHR monoclonal antibody (1:500; sc-137185, Santa Cruz Biotechnology, CA, USA) overnight at 4 °C and soaked in blocking buffer (1% BSA in PBS containing 0.1% Tween 20) for 1 h at room temperature. The membrane was briefly washed 3 times with PBS containing 0.1% Tween 20 and then incubated with secondary goat anti-mouse immunoglobulin-specific horseradish peroxidase-conjugated antibody (1:80 000; Sigma Aldrich, St. Louis, MO, USA) for 1 h at room temperature. After a 10-min washing step with PBS, the membrane was subjected to an enhanced chemiluminescence reaction using Pierce ECL Plus Western Blotting Substrate (Thermo Fisher Scientific) for 5 min before being exposed in the iBright FL1500 imaging system (Thermo Fisher Scientific). The blot was reprobed with mouse anti-β actin monoclonal horseradish peroxidase-conjugated antibody (1:100 000, sc-47778 HRP, Santa Cruz Biotechnology).

### Immunofluorescence staining

HepG2 transfected cells on cover slips were fixed with 10% neutral buffered formalin (Sigma-Aldrich, St. Louis, MO, USA) for 10 min before permeabilization with 0.2% Tween 20 (Duchefa, Haarlem, The Netherlands) in PBS (Vivantis, Selangor, Malaysia) for 30 min. This was followed by blocking with 10% Goat serum (Sigma-Aldrich) in PBST (PBS + 0.1% Tween 20) with 100 mM Glycine (Sigma-Aldrich) for 30 min. The cells were then incubated with primary mouse anti-GHR (B-10) monoclonal antibody (1:50; 0.2 mg/ml; sc-137185; Santa Cruz Biotechnology) at room temperature for 1 h. After rinsing 3 times with PBS, the cells were incubated with fluorescein conjugated goat anti-mouse antibody (1:500; 1 mg/ml; 710–1231; Rockland Immunochemicals, Limerick, PA, USA) for another 1 h at room temperature before mounting with mounting medium with DAPI (Vector Laboratories, Burlingame, CA, USA) and examination using the IX73 fluorescence microscope (Olympus, Tokyo, Japan).

### Packaging, purification, and titration of AAV8 Viral Vector

All AAV8 vector particles (single-stranded AAV8) were made by the 293T transient triple transfection method with HGT1, pAAV2-8 and AAV plasmid (pAAV-HLP-Luc or pAAV-HLP-mGHR in this study) as previously described [22]. Serotype 8 capsid pseudotyped viral particles were purified by the previously described iodixanol density gradient method [23]. The extracted vector particles were further purified from iodixanol contamination and concentrated using Amicon Ultra 4 100 K filter device (Millipore, Billerica, MA, USA) with PBS before aliquoted in 50 µL per tube and stored in −80 °C. Vector particles were titrated by quantitative PCR as described previously [24].

### Animal work

All animal experiments were performed according to the guidelines and protocols approved by the Institutional Animal Care and Use Committee (IACUC) of the National University of Singapore (protocol no. BR18-0735 and R18-0750). Because homozygous Laron mice are poorly fertile, heterozygous matings were used. The male C57BL/6J GHR+/− (heterozygotes) breeders were bred with female C57BL/6JInv GHR+/+ (wildtype) mice (InVivos Pte. Ltd., Singapore) to produce more heterozygotes male and female C57BL/6J GHR+/− breeders. One male and two females of C57BL/6J GHR+/− breeders were further bred to obtain male and female C57BL/6J GHR−/− (Laron dwarf mice). All mice were maintained in the specific pathogen-free facility within the university. The mice were subjected to regular 12 h dark/light cycles and provided with *ad libitum* of normal feed and water unless otherwise stated. Throughout this study, 4–5 weeks old male and female Laron dwarf mice were used for treatment with 4 × 10^10^ vector genome per mouse (vg/mouse) of AAV8-HLP-mGHR via intraperitoneal route of vector administration. AAV8-HLP-Luc at same dose was also injected in control Laron dwarf mice to serve as negative vector control. Five to six mice were used in each treatment group. Depending on the numbers of Laron dwarf mice and its gender from each breeding pair, AAV8-HLP-Luc and AAV8-HLP-mGHR were randomly injected in equal number of Laron dwarf mice from the same parent whenever possible and caged in same cage. Each mouse was assigned with a specific code number for tracking purposes and ease of recording. Blood was obtained for serum preparation (aliquoted and stored in −80 °C) at predetermined endpoints via cardiac puncture under 2–3% isoflurane vaporized gas anesthesia prior to cervical dislocation euthanasia. Liver tissues were immediately harvested in RNAlater (Ambion, Austin, TX, USA) for organ preservation and stored at −80 °C. Femur lengths and organ weights for brain, heart, lung, liver, spleen, and kidney were also measured at endpoint. All mice in this study were healthy and survived till endpoint. Mice that became moribund or severely underweight due to birth defect were euthanized by hypoxia using carbon dioxide followed by cervical dislocation. All efforts were made to minimize suffering.

### Mouse GH, IGF1, IGFBP3 and ALS ELISA

Serum mouse GH, IGF1, IGFBP3 and ALS at endpoint were measured using ELISA kits for mouse GH (CSB-E07343m CUSABIO, Wuhan, Hubei Province, China), mouse IGF1 (EMIGF1; Thermo Fisher Scientific), mouse IGFBP3 (MGB300; R&D systems Minneapolis, MN, USA) and mouse IGFALS (CSB-EL011094MO; CUSABIO), respectively according to manufacturing instructions. Molar ratio of mouse IGF1 to IGFBP3 was calculated as previously described [25] using the formula, ratio = [IGF1 (ng/mL) × 0.1307]/[IGFBP3 (ng/mL) × 0.03478] = IGF1 (nmol/L)/IGFBP3 (nmol/L).

### RT-PCR of mouse GHR, IGF1, IGFBP3 and ALS mRNA transcript levels in the mouse livers

Total RNA from the livers of treated mice was isolated using the AllPrep DNA/RNA Mini Kit (QIAGEN), according to the manufacturer’s instructions. One µg of total RNA was converted to cDNA using random hexamer primers (Integrated DNA Technologies), oligo-dT primers (Integrated DNA Technologies) and RNase H Minus Reverse Transcriptase (Thermo Fisher Scientific) according to the manufacturer’s protocol. Relative mRNA expression in the liver was quantitated by real-time qPCR assay using Roto-Gene 3000 (Corbett Research, Sydney, Australia) and Rotor-Gene SYBR Green PCR Kit (QIAGEN). The primers were designed against the mouse GHR [21] (forward primer 5’-GATTTTACCCCCAGTCCCAGTTC-3’ and reverse primer 5’-GACCCTTCAGTCTTCTCATCCACA-3’), mouse IGF1 [21] (forward primer 5’-GTGTGGACCGAGGGGCTTTTACTTC-3’ and reverse primer 5’-GCTTCAGTGGGGCACAGTACATCTC-3’), mouse IGFBP3 [21] (forward primer 5’-AAGTTCCATCCACTCCATGC-3’ and reverse primer 5’-TTCTGGGTGTCTGTGCTTTG-3’), mouse ALS [21] (forward primer 5’-AGCTCAGCGTCTTTTGCAGT-3’ and reverse primer 5’-ACAGGTTGTTTCCGTCAAGC-3’) and normalized against mouse 18S rRNA (forward primer 5’-CCTGCGGCTTAATTTGACTC-3’ and reverse primer 5’-CGCTGAGCCAGTCAGTGTAG-3’). All PCR reactions were performed in duplicate. The CT of each sample was obtained using RotorGene version 6.1 software. 2^-(ΔΔCT)^ was calculated and the relative expression levels were calculated by arbitrarily designating a value to one.

### AAV viral genome copies quantification

Total genomic DNA from the livers of treated mice was extracted using AllPrep DNA/RNA mini kit according to manufacturing protocol (QIAGEN). AAV viral genome copy number was determined by real-time qPCR using Rotor-Gene 3000 (Corbett Research, Sydney, Australia) and Rotor-Gene SYBR Green PCR Kit (QIAGEN). The primers were designed against the HLP promoter and normalized with mouse GAPDH housekeeping gene to calculate AAV genome copies/µg of DNA.

### Statistical analyses

The number of animals used in each group is indicated in the respective figure legends. All values are expressed as mean ± s.e.m. Statistical significance for studies involving two groups were determined by Student’s unpaired *t*-test whereas statistical significance for studies involving more than two factors were determined by Two-way ANOVA where ns indicated not significant, *indicates *p* < 0.05 and considered statistically significant, **indicates *p* < 0.01 and considered very significant, ***indicates *p* < 0.001 and considered extremely significant and ****indicates *p* < 0.0001 and considered extremely significant. All statistically compared groups had similar variance as analyzed using Brown–Forsythe test.

## Results

### Creation of AAV plasmid expressing mGHR for treatment of Laron dwarf mice

**Fig. 1:**
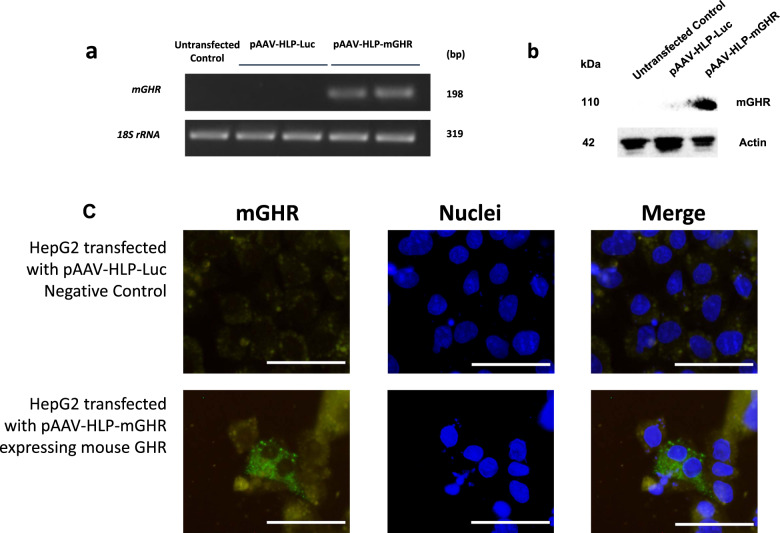
In vitro expression of mouse growth hormone receptor (mGHR) in HepG2 human liver cancer cell line. **a** RT-PCR gel of mGHR mRNA expression. 18S rRNA housekeeping gene was used as loading control. **b** Western blot of mGHR protein expression. Actin housekeeping gene was used as loading control. **c** Immunofluorescence staining of mGHR expression, scale bars are 50 µm and nuclei were stained with DAPI. HepG2 cells were transfected with pAAV-HLP-mGHR consisted of hybrid liver-specific promoter (HLP) driving the expression of mGHR. Untransfected control and cells transfected with pAAV-HLP-Luc expressing luciferase gene were used as negative control. Analyses were performed 48 h post-transfection.

We have previously generated a hepatocyte-specific AAV8 vector that specifically expressed the human insulin gene in the liver only. Expression with this liver-specific hybrid liver-specific promoter (HLP) [26] was absent in all other tissues and cells. Upon systemic delivery, the AAV8 vector successfully showed treatment efficacy and potentially served as future long-term basal insulin gene therapy for diabetes patients [19, 27, 28]. In this study, we further explored the potential of AAV8 vector to express mGHR regulated by the same liver-specific HLP promoter for treatment of Laron dwarf mice. The mRNA and protein expression of mGHR encoded by pAAV-HLP-mGHR was confirmed in vitro by transfecting the vector into HepG2 cells as shown in Fig. 1a and Fig. 1b, respectively. Immunofluorescence staining (Fig. 1c) indicated that mGHR expression was localized to the plasma membrane, cytosol, and cytoplasmic bodies of HepG2 cells similar to our previous observations [29].

### Liver expression of mGHR conferred by AAV8 viral vector significantly increases body weight and length of Laron dwarf mice in both male and female

**Fig. 2:**
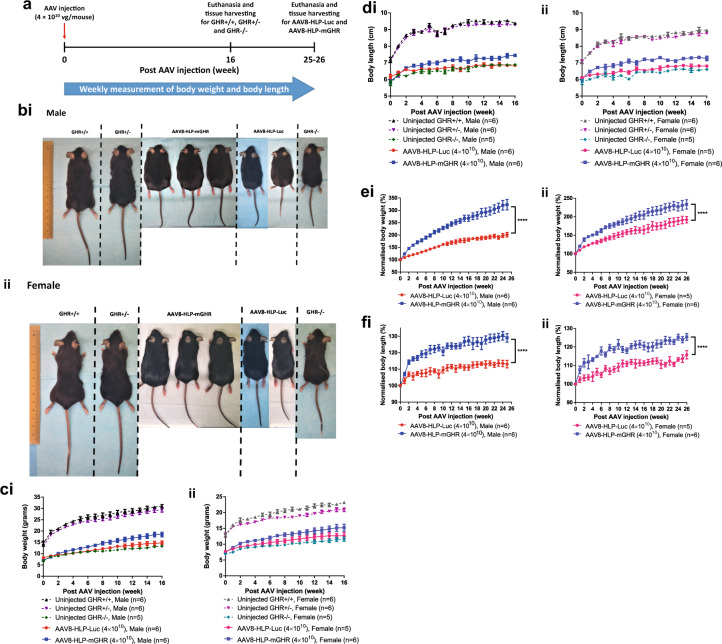
In vivo treatment efficacy of AAV8-HLP-mGHR in Laron mice. **a** Timeline summarized the experiment performed. **b** Representative photos for uninjected control mice (GHR+/+, GHR+/− and GHR−/−) and Laron mice (GHR−/−) injected with 4 × 10^10^ vg/mouse of AAV8-HLP-Luc and AAV8-HLP-mGHR in male (i) and female (ii). Original unmodified image was shown in Supplementary Fig. S1. Weekly measurement of (**c**) body weight and (**d**) body length post AAV injection and the corresponding (**e**) normalized body weight and (**f**) normalized body length. Body weight and length were normalized to 100% at the time of AAV injection to correct the initial body weights and length variation of mice. AAV8-HLP-mGHR and AAV8-HLP-Luc treated groups were monitored for 25–26 weeks post AAV injection while uninjected control groups were monitored for 16 weeks only. Data were presented as mean ± s.e.m. *n* = 5–6. Statistical significances were determined by two-way ANOVA where **** indicates *p* < 0.0001 and considered extremely significant.

To evaluate the in vivo treatment efficacy of this construct, pAAV-HLP-mGHR was packaged into AAV8-HLP-mGHR viral vector and intraperitoneally (i.p.) injected into 4 to 5-week-old Laron dwarf mice at dose of 4 × 10^10^ vector genome per mouse (vg/mouse) according to experiment timeline as indicated in Fig. 2a. AAV8-HLP-Luc was also injected into Laron mice to serve as negative controls. Untreated wildtype (GHR+/+), heterozygous (GHR+/−) and Laron (GHR−/−) dwarf mice were included for comparisons.

As shown in the photos of representative mice in Fig. 2bi (male) and ii (female), AAV8-HLP-mGHR treated male and female Laron mice were larger in size and weighed more when compared to their respective gender untreated Laron (GHR−/−) and AAV8-HLP-Luc injected controls. However, the AAV8-HLP-mGHR treatment only partially restored the treated mice towards the normal size of GHR+/+ mice. The body weight and length for male and female mice from the time of AAV injection were shown in Fig. 2ci-ii and Fig. 2d i-ii, respectively. The wildtype and heterozygous mice showed rapid growth in weight and body length for the initial 8 weeks of experiment and the growth slowed down or plateaued respectively after 8 weeks. The body weight and length of Laron (GHR−/−) controls and treated mice increased gradually throughout the experiment.

Table 1 summarizes the percentage of body weight (calculated from Fig. 2ci–ii) and length (from Fig. 2di-ii) normalized to GHR+/+ ’s body weight and length (100%) at 16-week post AAV injection (equivalent to 20–21-week-old of mice age). The body weight of male Laron mice and Laron mice treated with control AAV8-HLP-Luc was 42.9 ± 1.2 and 47.7 ± 2.6% of GHR+/+, respectively, and increased to 59.5% compared to wildtype upon AAV8-HLP-mGHR treatment. The body length of AAV8-HLP-mGHR treated Laron mice reached 78.7 ± 0.8% of GHR+/+. No difference in body length was observed between AAV8-HLP-Luc injected and untreated mice. Similar findings were observed in female mice with slightly higher in percentage of GHR+/+ due to smaller normal physiological size of GHR+/+ female mice when compared to male mice. The average body weight of AAV8-HLP-mGHR treated female Laron mice was higher than AAV8-HLP-Luc injected mice, but it showed inconsistent responses which led to a non-significant difference between the groups.

**Table 1:** Percentage of body weight and body length when compared to wildtype mice at corresponding sex and age.

	Percentage of normal wildtype as 100% (at 16-week post AAV injection or equivalent to 20 to 21-week-old of mice age)									
	Uninjected control						AAV8 dose: 4 × 10^10^ vg/mouse			
	GHR+/+		GHR+/−		GHR−/−		AAV8-HLP-Luc		AAV8-HLP-mGHR	
	Mean	±SEM	Mean	±SEM	Mean	±SEM	Mean	±SEM	Mean	±SEM
Male										
Body weight	100.0	2.3	94.6	2.9	42.9	1.2	47.7	2.6	59.5*	3.3
Body length	100.0	1.0	98.9	1.0	73.3	0.5	73.0	0.9	78.7***	0.8
Female										
Body weight	100.0	1.2	89.9	2.4	50.1	2.7	54.8	4.1	65.6n.s.	4.6
Body length	100.0	1.1	98.0	0.9	73.7	0.6	76.0	0.9	81.1*	1.5

n.s. *P* > 0.05; *P < 0.05; ***P < 0.001 Student’s unpaired *t*-test comparing values between AAV8-HLP-Luc and AAV8-HLP-mGHR.

To accurately compare the treatment efficacy, the body weight and length were normalized to 100% at the time of AAV injection and measurements were standardized to baseline until 25 to 26-week post AAV injection as shown in Fig. 2e (body weight) and Fig. 2f (body length) for male (i) and female (ii) treated mice. Two-way ANOVA analysis revealed that the differences between body weight and length regardless of gender were highly significant in AAV8-HLP-mGHR treated Laron mice when compared to vector control AAV8-HLP-Luc injected Laron mice. These observations confirmed the functionality and capability of AAV8-HLP-mGHR vector in improving the size of Laron dwarf mice. Nevertheless, it is important to note that the increase in body weight sizes were mainly driven by obesity especially in treated male Laron dwarf mice as can be seen in Fig. 2bi, while only moderate increase in the growth of body lengths were observed.

### In vivo functionality of AAV8-mediated expression of mGHR as indicated by GH normalization, increased IGF1, ALS, IGFBP3, femur length and organ weights

**Fig. 3:**
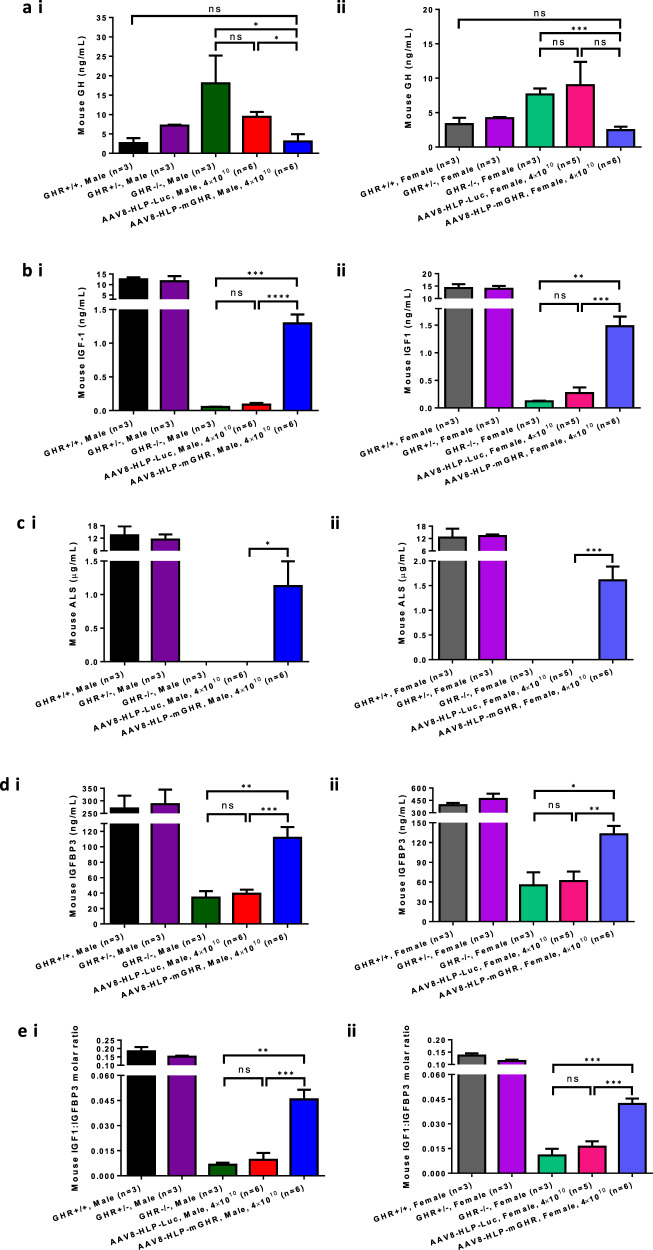
Circulating GH, IGF1, ALS and IGFBP3 of AAV8-HLP-mGHR treated Laron mice. Serum level of mouse (**a**) GH, (**b**) IGF1, (**c**) ALS, (**d**) IGFBP3 and (**e**) IGF1:IGFBP3 molar ratio at end point in (i) male and (ii) female of uninjected GHR+/+, GHR+/−, GHR−/− and AAV-injected Laron dwarf mice with AAV8-HLP-Luc and AAV8-HLP-mGHR. Data were presented as mean ± s.e.m. *n* = 3–6. Statistical significances were determined by Student’s unpaired *t*-test where ns indicated not significant, * indicates *p* < 0.05 and considered statistically significant, ** indicates *p* < 0.01 and considered very significant, *** indicates *p* < 0.001 and considered extremely significant and **** indicates *p* < 0.0001 and considered extremely significant.

To elucidate the changes of GH and its targets accompanying the expression of AAV8-HLP-mGHR in Laron mice, blood was collected at endpoint (32- to 34-week-old) and mice sera were prepared for ELISA quantification of GH, IGF1, ALS and IGFBP3 levels. The elevated serum level of GH found in Laron mice was decreased significantly compared to the level of the wildtype mice with AAV8-HLP-mGHR treatment (Fig. 3ai–ii). Accordingly, the serum level of IGF1 was increased in AAV8-HLP-mGHR treated Laron mice when compared to untreated (GHR−/−) and AAV8-HLP-Luc treated mice (Fig. 3bi–ii). The mouse IGF1 levels of male GHR+/+ and GHR+/− mice were 12.47 ± 1.00 and 11.55 ± 2.45 ng/ml, respectively, whereas the GHR−/− and AAV8-HLP-Luc treated mice expressed very low and near undetectable levels of IGF1 at 0.055 ± 0.004 ng/ml and 0.088 ± 0.026 ng/ml, respectively. The AAV8-HLP-mGHR treatment successfully increased the IGF1 level significantly higher than GHR−/− mice, although the level was only 10% of the GHR+/+ mice. The female wildtype showed the same trend with an increase in IGF1 in the serum and incomplete restoration to that of the GHR+/+ mice. Similar responses were observed in ALS and IGFBP3 as shown in Fig. 3ci-ii and Fig. 3di-ii, respectively. The changes in ALS were especially obvious in both male (1.126 ± 0.370 µg/ml) and female (1.607 ± 0.281 µg/ml) for AAV8-HLP-mGHR treated Laron mice when compared to undetected levels in corresponding untreated and AAV8-HLP-Luc treated groups. Molar ratio of mouse IGF1 to IGFBP3 further confirmed the functionality of AAV8-HLP-mGHR treatment as shown in Fig. 3ei-ii. These results differ significantly from the human injections of recombinant IGF1 where there is no increase in ALS [30].

**Fig. 4:**
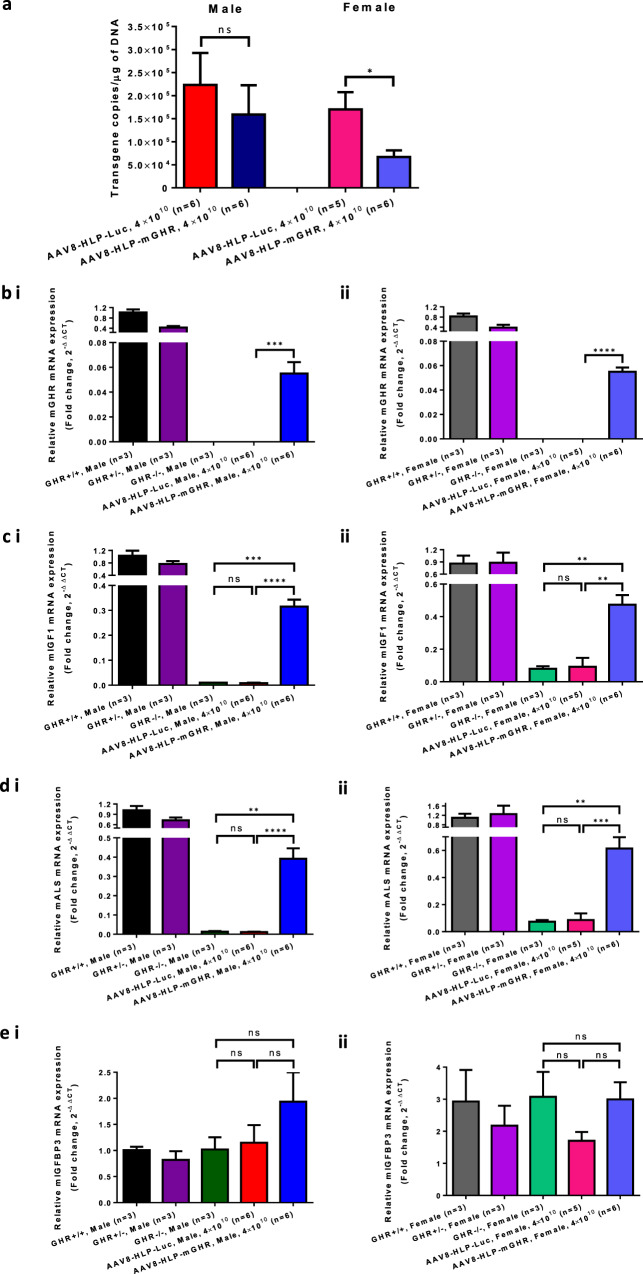
Confirmation of viral gene delivery to liver and its effect on mouse transcript of GHR, IGF1, ALS and IGFBP3. **a** Quantification of viral transgene copies number in mouse liver. The copies numbers were normalized with mouse GAPDH housekeeping gene. Messenger RNA expression of mouse (**b**) GHR, (**c**) IGF1, (**d**) ALS and (**e**) IGFBP3 at end point in (i) male and (ii) female of uninjected GHR+/+, GHR+/−, GHR−/− and AAV-injected Laron dwarf mice with AAV8-HLP-Luc and AAV8-HLP-mGHR. The mRNA expression levels were normalized with 18S rRNA housekeeping gene. Data were presented as mean ± s.e.m. *n* = 3–6. Statistical significances were determined by Student’s unpaired *t*-test where ns indicated not significant, * indicates *p* < 0.05 and considered statistically significant, ** indicates *p* < 0.01 and considered very significant, *** indicates *p* < 0.001 and considered extremely significant and **** indicates *p* < 0.0001 and considered extremely significant.

Measurement of hepatic viral genome copy number (Fig. 4a) revealed lower transgene copies per µg of DNA in AAV8-HLP-mGHR in the female treated group when compared to female Laron mice injected with AAV8-HLP-Luc. This was probably due to a dilution effect of non-replicative episomal AAV genome [16, 31, 32] when liver size increased with AAV8-HLP-mGHR treatment and not with AAV8-HLP-Luc.

RT-PCR analysis for liver mRNA expression of mouse GHR (Fig. 4bi-ii) confirmed the in vivo liver expression of GHR in AAV8-HLP-mGHR injected Laron mice. The increased serum level of circulating IGF1 and ALS (see Fig. 3bi-ii and Fig. 3ci-ii) correlated well with the increment of mRNA expression of IGF1 (Fig. 4ci-ii) and ALS (Fig. 4di-ii) in mouse liver. However, no significant change in IGFBP3 (Fig. 4ei-ii) mRNA transcripts was observed. The increase in serum level of IGFBP3 (refer to Fig. 3di-ii) could be due to improved serum stability of IGFBP3 as a result of forming ternary complexes with increased serum level of IGF1 and ALS.

**Fig. 5:**
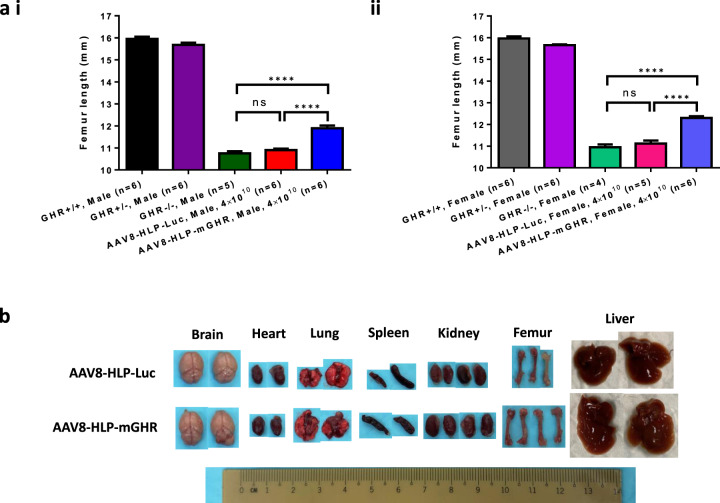
Effect of AAV8-HLP-mGHR treatment on femurs length and organs sizes of Laron dwarf mice. **a** Femur length in (i) male and (ii) female of uninjected GHR+/+, GHR+/−, GHR−/− and AAV-injected Laron dwarf mice with AAV8-HLP-Luc and AAV8-HLP-mGHR. Data were presented as mean ± s.e.m. *n* = 4–6. Statistical significances were determined by Student’s unpaired *t*-test where ns indicated not significant and **** indicates *p* < 0.0001 and considered extremely significant. **b** Photo of organs and femurs of AAV8-HLP-Luc and AAV8-HLP-mGHR injected Laron dwarf mice harvested at endpoint. Original unmodified image was shown in Supplementary Fig. S1.

Elevated serum levels of circulating IGF1, ALS and IGFBP3 resulted in a significant increase in Laron mice femur length (Fig. 5a) in both male (i) and female (ii). No significant difference in femur length between GHR−/− and AAV8-HLP-Luc negative control was observed. Liver and kidney were clearly larger in AAV8-HLP-mGHR treated Laron mice when compared to AAV8-HLP-Luc controls (Fig. 5b). Weights of spleen, lung and heart were also significantly increased but the differences were small. Interestingly, no significant change was observed in brain weight. The organ weights of all experimental mice are summarized in Table 2.

**Table 2:** Organ weights of mice at end point.

	Organ weight (mg)									
	Uninjected control						AAV8 dose: 4 × 10^10^ vg/mouse			
	GHR+/+		GHR+/−		GHR−/−		AAV8-HLP-Luc		AAV8-HLP-mGHR	
Organ	Mean	±SEM	Mean	±SEM	Mean	±SEM	Mean	±SEM	Mean	±SEM
Male										
Liver	1466.67	66.67	1376.67	78.39	380.00	20.00	483.33	30.73	670.00**	49.67
Brain	476.43	5.61	476.97	10.41	395.08	6.52	407.90	7.67	402.78	5.35
Kidney	421.90	29.32	398.72	25.81	128.46	11.07	163.45	13.53	249.10**	14.58
Spleen	95.08	6.03	97.13	5.64	30.56	3.03	36.78	3.15	51.65*	5.68
Heart	184.25	10.59	177.12	7.97	67.04	3.51	80.45	4.87	94.03	5.78
Lung	185.33	3.43	173.68	7.97	80.46	6.18	93.95	6.43	116.30*	5.07
Female										
Liver	966.67	33.33	950.00	71.88	425.00	47.87	400.00	31.62	550.00**	22.36
Brain	493.35	16.61	496.05	8.55	403.53	7.41	402.86	3.63	418.05	9.88
Kidney	300.05	9.25	305.32	13.80	117.93	5.40	132.68	10.92	193.55**	7.45
Spleen	102.58	9.14	91.28	5.46	31.85	1.30	38.30	3.19	55.42**	3.86
Heart	136.67	2.56	126.92	3.35	65.98	2.79	65.52	2.25	77.33**	2.66
Lung	166.20	7.89	240.25	85.22	79.83	4.90	86.54	4.43	100.90*	3.96

*P < 0.05, **P < 0.01. Student’s unpaired t-test compared to mice treated with AAV8-HLP-Luc.

## Discussion

Gene therapy using an AAV viral vector has emerged as a safe and efficient gene delivery tool with the potential to treat a variety of inherited or rare mutation disorders that previously were untreatable, such as hemophilia, Duchenne muscular dystrophy (DMD), spinal muscular atrophy (SMA), Pompe disease etc. (see review by Wang and colleagues for more details [16]). Here, we have successfully demonstrated for the first time that the AAV vector can be used to treat Laron dwarfism with a single AAV injection dose using a mouse model with disruption of the mGHR gene [18]. This would compare favorably to multiple rhIGF1 injections (once or twice daily) that have several limitations [11] including the pain and discomfort to patients. Notably, liver mGHR expression conferred by AAV8 viral vector achieved statistically significant efficacy in both male and female Laron dwarf mice evidenced by significant increases in body weight and body length.

Our findings showed that a single intraperitoneal dose of AAV8-HLP-mGHR (4 × 10^10^ vg/mouse) into 4–5-week-old Laron dwarf mice resulted in sufficient expression of mGHR in the liver to restore GH signaling resulting in increased circulating IGF1, IGFBP3 and ALS. This is significantly different from injections of rhIGF1 in human Laron syndrome with absence of GH dependent IGFBP3 and ALS after treatment [30]. The formation of the ternary IGF complex with IGFBP3 and ALS has been reported to be crucial for postnatal bone acquisition and skeletal response [33]. The formation of ternary IGF complex would further stabilize the serum IGF1 and this might be a possible reason for the significant lowering of the high serum GH level in Laron dwarf mice as shown in Fig. 3a. Interestingly, full suppression of GH levels was observed with only partially elevated IGF1. Our data suggests that the IGF1 feedback on pituitary GH secretion is more sensitive (inhibitory) than the hepatic IGF1 secretory response to GH via the expressed GH receptor. It may be that the virtual absence of circulating IGF1 has resulted in sensitization of the IGF1 sensor in the pituitary so that GH secretion is preferentially inhibited. Further studies may be needed to elucidate this.

The mean body weight increase at 25-week post AAV injection (i.e., 29–30-week-old mice) was 34% (male) and 16% (female) in AAV8-HLP-mGHR treated mice as compared to control mice treated with AAV8-HLP-Luc. The corresponding mean body length increase was 8.4% (male) and 7.4% (female), respectively. Our treatment efficacy was comparable to the results reported by Luca and colleagues who used microencapsulated IGF1-expressing porcine Sertoli cells to treat the same Laron mouse model which produced a similarly limited 30% increase in body weight and 9% increase in body length [34]. These observations suggested that liver expression of mGHR may restore the liver expression of circulating IGF1 comparable to IGF1 treatment. Our system may have an advantage compared to transplanted microencapsulated cells with its known difficulties of oxygenation and potential immune rejection.

After long-term daily injections of rhIGF1, some patients successfully reach normal 3^rd^ percentile height of about 155 cm [35]. However, for most treated patients with LS, the mean increase is only 13.4 cm, ranging from 10 to 15 cm [11] (i.e., 74–80% height of normal 50^th^ percentile of 175 cm). Our treatment of Laron mice resulted in a similar partial restoration of body length (Fig. 2d) where the AAV8-HLP-mGHR treated mice had grown at near normal rates after 3-week post AAV injection but did not display catch up growth to normal. Therefore, it is unlikely that treatment of Laron mice at our dosage of viral particles could reach regular adult size. The present outcomes are thus limited, and were probably related to the decrease of mouse serum IGF1 concentration after its peak at 7 weeks of age [36] similar to the reduced human growth rate after peak IGF1 during puberty [37]. Further refinement of our treatment may thus be needed. There are at least two possibilities – a single injection with a higher dose of AAV8-HLP-mGHR or a repeated injection at an older age.

Another possibility for the limitations in efficacy seen in our study is that IGF1 induced growth alone without synergistic GHR activity locally in other tissues may not give full growth. This has been suggested by a study in a transgenic mouse model that found limited IGF1 expression in liver resulting in only about 50% of GHR+/+ body weight [38]. Local autocrine or paracrine IGF1 expression induced by local GHR action may be necessary to assist in the bone lengthening process [39–41].

Other than the limited increase in body length, Laron dwarf mice, especially in males, treated with AAV8-HLP-mGHR also suffered from obesity as shown in Fig. 2b. The increase in body weight of these mice were partly due to accumulation of visceral and subcutaneous adipose tissues evident during experiments presumably via the IGF1 lipogenic effect [42] in the absence of GH action in adipocytes. Although the expression of mGHR in liver was restored with AAV8-HLP-mGHR in Laron dwarf mice, mGHR expression is probably also required in adipose tissue to prevent obesity as studies have found that FaGHRKO (with selectively disrupted GHR in adipose tissue) mice showed an obese phenotype with increased total fat and increased adipocyte size [43, 44]. GHR expression driven by a ubiquitous promoter may resolve this issue, or targeted GHR expression based on results with tissue-specific GHR knockout mice [45] may help to design a more specific treatment strategy.

We also observed in Fig. 4e. that there was no significant difference in IGFBP3 mRNA transcript between GHR+/+ and GHR−/− mice. This did not directly correlate with the circulating mIGFBP3 level that was significantly lower in untreated Laron mice when compared to GHR+/+ and AAV8-HLP-mGHR treated controls as shown in Fig. 3d. This result suggests that mIGFBP3 mRNA expression may be constitutive, being similar in both GHR−/− and treated animals, but the expressed mIGFBP3 might not be stable. mIGFBP3 is stabilized through formation of a ternary complex with mIGF1 and mALS that are expressed upon activation of mGHR, see Figs. 3b, c and 4c, d. A similar phenomenon was also observed in the Epha4(−/−) knockout mouse. The EphA4 gene deleted mouse had impaired STAT5B activation (downstream target of GH/GHR activation pathway [46]) that led to reduced IGF1 expression [21]. Overall, the data suggested that AAV8-HLP-mGHR treatment may have potential to mimic the effectiveness of rhIGF1 [4–6, 47].

Taken together, we have successfully demonstrated as a proof of concept that gene therapy of Laron dwarf mice produces an efficacy similar to the rhIGF1 treatment in humans [11] or xenotransplantation of IGF1-expressing porcine cells [34] (summarized in Supplementary Table S1). However, for successful treatment of LS, further improvement is needed to express mGHR to extra-hepatic tissues using ubiquitous promoters/enhancers such as CAG (CMV enhancer/chicken beta Actin promoter/rabbit beta-Globin intron) promoter to target its expression to more tissues, especially to adipose tissue to prevent obesity [44], as well as growth plates for normal elongation of the bone [39]. In addition, earlier treatment of Laron dwarf mice may be necessary because mice reach an early IGF1 peak at around 7 weeks of age [36]. Higher AAV dose may be also useful to improve the treatment efficacy since the current dose of 4 × 10^10^ vg/mouse only expressed about 5% of GHR that is normally produced in wildtype mice (Fig. 4b). However, we are cautious about increasing the AAV dose due to potential genomic integration events [48]. When targeting to proliferating cells such as growth plate, AAV may not be a good candidate as a gene delivery tool due to its non-replicative nature and will be diluted out upon cell proliferation [49]. Finally, introduction of a cytoplasmic domain SNP which impairs SOCS2 binding and hence extends receptor signaling life could partly compensate for the lower receptor expression [50]. Despite its limitations, this study provides evidence that single administration of AAV8-HLP-mGHR viral vector can increase the expression of endogenously circulating IGF1, IGFBP3 and ALS and potentially lead to a useful treatment for patients with LS.

## Supplementary information

Supplementary Fig. S1. Original unmodified images with relevant rulers for Figure 2ai, 2aii and 5b.

Supplementary Table S1. Summary on AAV8-HLP-mGHR mouse gene therapy compared to other treatments with human IGF1 discussed in Discussion.
