# Supplementary material for: First use of gene therapy to treat growth hormone resistant dwarfism in a mouse model
Source: Gene Ther. 2022 Feb 1;29(6):346–56. doi: 10.1038/s41434-022-00313-w (PMC9203273; doi:10.1038/s41434-022-00313-w)
Supplement: Supplementary file 1 — Supplementary Fig. S1. Original unmodified images with relevant rulers for Figure 2ai, 2aii and 5b. [file 41434_2022_313_MOESM1_ESM.pptx]

## Slide 1
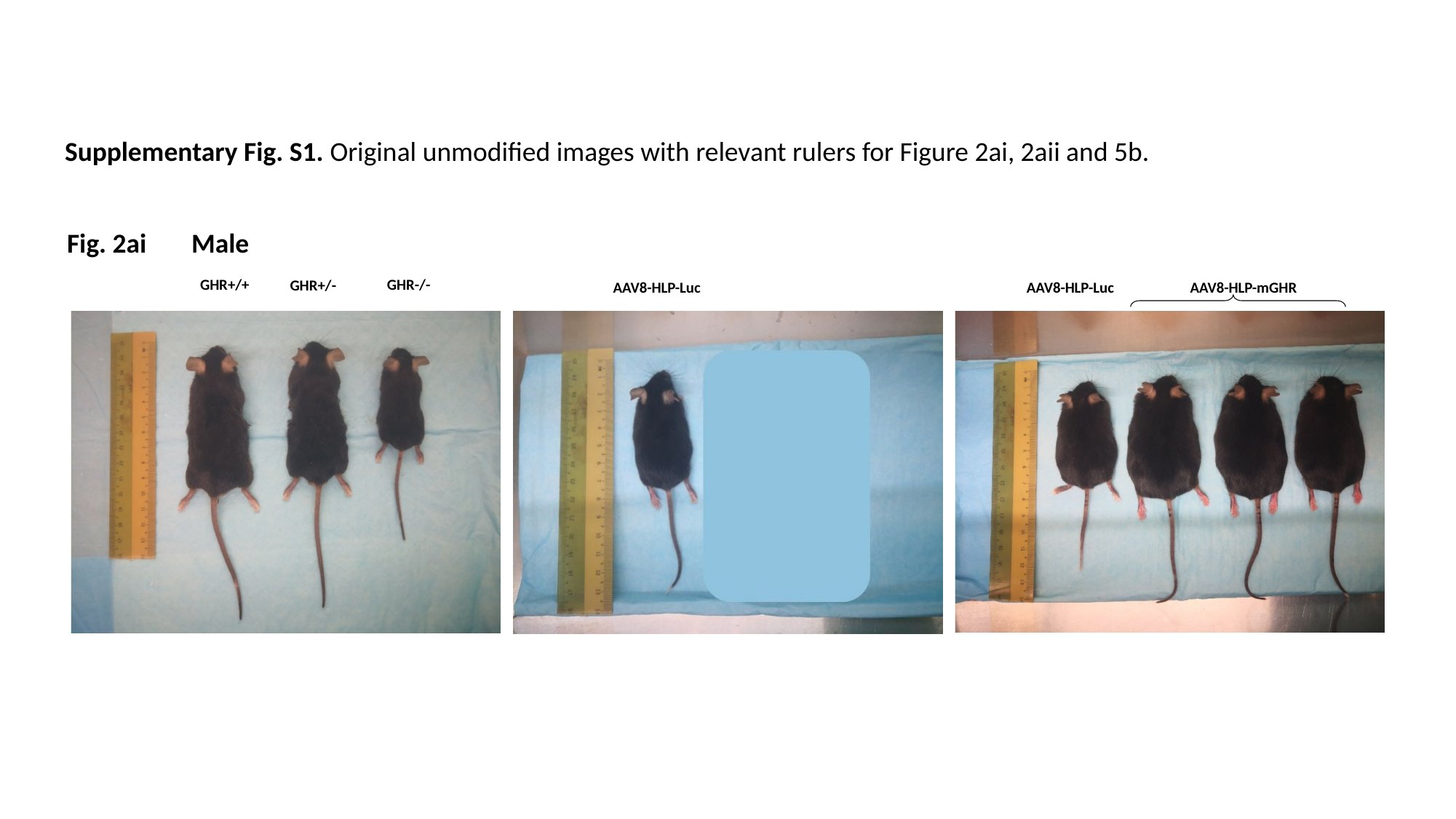

Supplementary Fig. S1. Original unmodified images with relevant rulers for Figure 2ai, 2aii and 5b.
Fig. 2ai
Male
GHR+/+
GHR+/-
GHR-/-
AAV8-HLP-Luc
AAV8-HLP-Luc
AAV8-HLP-mGHR

## Slide 2
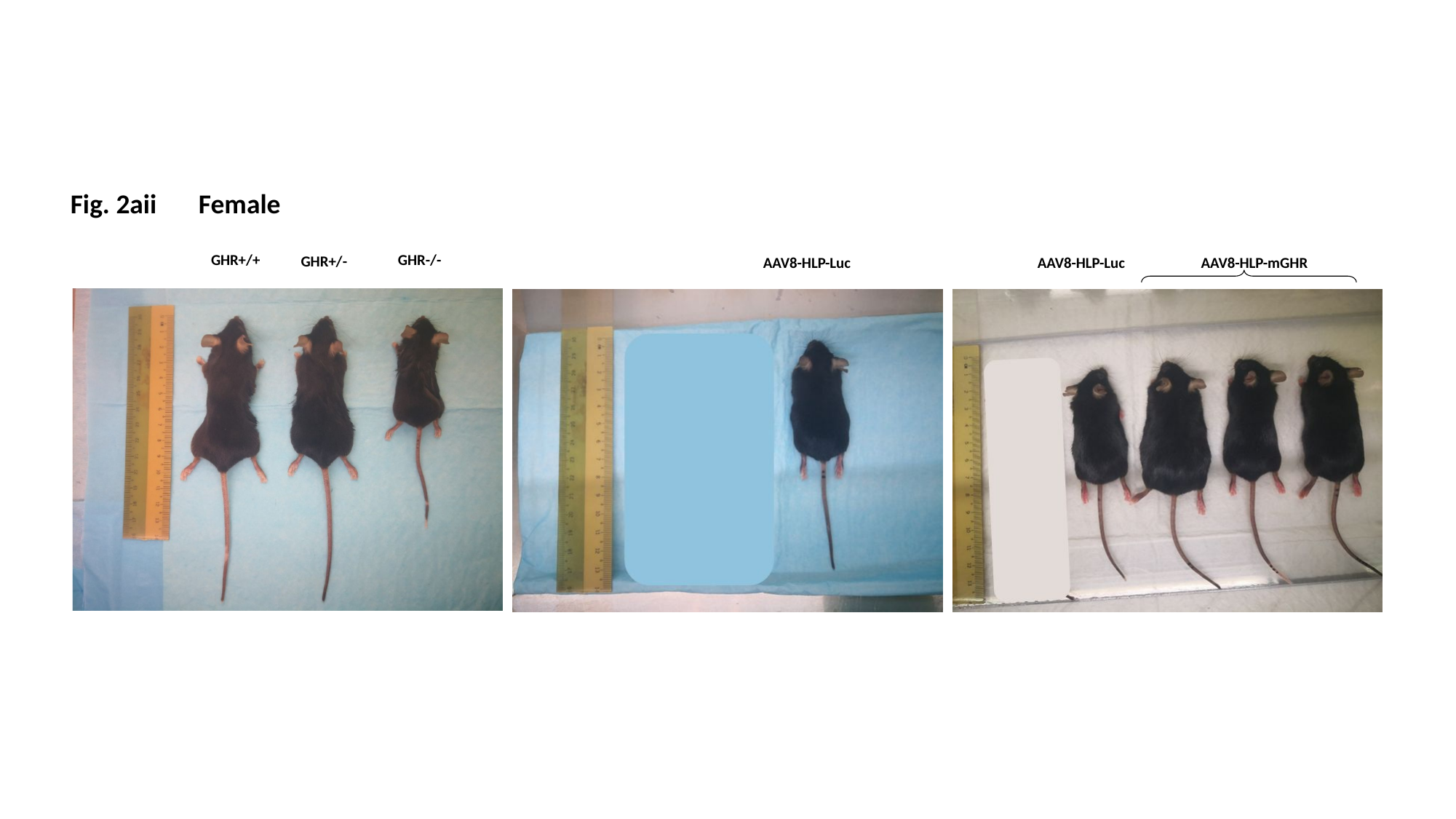

Fig. 2aii
Female
GHR+/+
GHR+/-
GHR-/-
AAV8-HLP-Luc
AAV8-HLP-Luc
AAV8-HLP-mGHR

## Slide 3
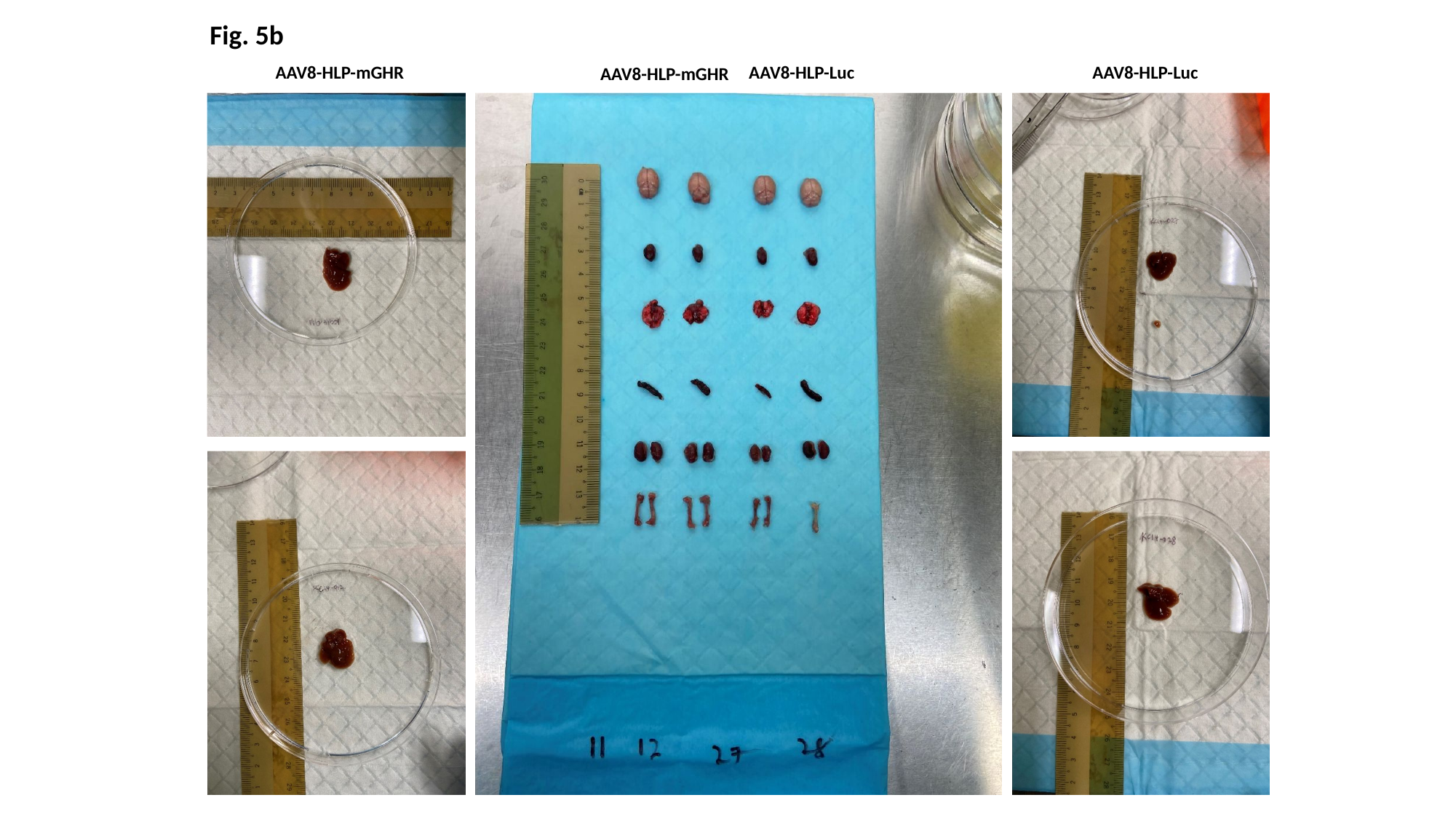

Fig. 5b
AAV8-HLP-mGHR
AAV8-HLP-mGHR
AAV8-HLP-Luc
AAV8-HLP-Luc
