# Supplementary material for: First use of gene therapy to treat growth hormone resistant dwarfism in a mouse model
Source: Gene Ther. 2022 Feb 1;29(6):346–56. doi: 10.1038/s41434-022-00313-w (PMC9203273; doi:10.1038/s41434-022-00313-w)
Supplement: Supplementary file 2 — Supplementary Table S1. Summary on AAV8-HLP-mGHR mouse gene therapy compared to other treatments with human IGF1 discussed in Discussion. [file 41434_2022_313_MOESM2_ESM.docx]

| **Treatment (Duration)** |  | **Dose** |  | **Subject (age)** |  | **Efficacy** | | |  | **Side Effects** |  | **Reference** |  |
| --- | --- | --- | --- | --- | --- | --- | --- | --- | --- | --- | --- | --- | --- |
| **In vivo** |  |  |  |  |  | **Body Length increase** |  | **Body Weight increase** |  |  |  |  | |
| AAV8-HLP-mGHR |  | 4 × 10^10^ vg/mouse (single dose) |  | Laron Dwarf Mouse  (4-5 weeks old) |  | Male : 8.4%  Female: 7.4%  (29-30 weeks old) |  | Male : 34%  Female: 16%  (29-30 weeks old) |  | Obesity |  | This study | |
|  |  |  |  |  |  |  |  |  |  |  |  |  | |
| Microencapsulated IGF1-expressing porcine Sertoli cells (pSC) |  | 1 × 10^6^ pSC/g (single dose) |  | Laron Dwarf Mouse  (10 days old) |  | 9%  (24 weeks old) |  | 30%  (24 weeks old) |  | Obesity |  | [34] | |
|  |  |  |  |  |  |  |  |  |  |  |  |  | |
| **Clinical Trials** |  |  |  |  |  | **Body Height^*^** |  | **Body Weight^*^** |  |  |  |  | |
| rhIGF1 (4 to 13 years) |  | 75-250 μg/kg q.d. or 40-120 μg/kg b.i.d. |  | Children with Laron Syndrome  (7 months – 19 years old) |  | Mean height gain 13.4 cm (10-15 cm) |  | nil |  | Progressive obesity, hypoglycemia, lipohypertrophy, coarsening of facial features, increase in melanocytic nevi, mild LDH elevation, snoring, hypoacusis, hyperandrogenism and intracranial hypertension. |  | [11] | |
|  |  |  |  |  |  |  |  |  |  |  |  |  | |
| rhIGF1 only or with IGFBP3  (15 years) |  | Mean 200 μg IGF1/kg/day  (q.d. or b.i.d.) |  | Child AA with Laron Syndrome  (5 years old) |  | 155.7 cm  (20.3 years old) |  | 60.6 kg  (20.3 years old) |  | Hypoglycaemia and  lipohypertrophy |  | [35] | |

**Supplementary Table S1**. Summary on AAV8-HLP-mGHR mouse gene therapy compared to other treatments with human IGF1 discussed in Discussion.

*Final values after end of the clinical trials, q.d. – once daily, b.i.d. – twice daily
